# Photodynamic Therapy Based on Graphene and MXene in Cancer Theranostics

**DOI:** 10.3389/fbioe.2019.00295

**Published:** 2019-10-25

**Authors:** Arianna Gazzi, Laura Fusco, Anooshay Khan, Davide Bedognetti, Barbara Zavan, Flavia Vitale, Acelya Yilmazer, Lucia Gemma Delogu

**Affiliations:** ^1^Department of Chemical and Pharmaceutical Sciences, University of Trieste, Trieste, Italy; ^2^Fondazione Istituto di Ricerca Pediatrica Città della Speranza, Padua, Italy; ^3^Sidra Medical and Research Center, Doha, Qatar; ^4^Department of Biomedical Engineering, University of Ankara, Ankara, Turkey; ^5^Department of Medical Sciences, University of Ferrara, Ferrara, Italy; ^6^Maria Cecilia Hospital, GVM Care & Research, Ravenna, Italy; ^7^Department of Neurology, Bioengineering, Physical Medicine & Rehabilitation, Center for Neuroengineering and Therapeutics, University of Pennsylvania, Philadelphia, PA, United States; ^8^Center for Neurotrauma, Neurodegeneration, and Restoration, Corporal Michael J. Crescenz Veterans Affairs Medical Center, Philadelphia, PA, United States; ^9^Stem Cell Institute, University of Ankara, Ankara, Turkey; ^10^Department of Biomedical Sciences, University of Padua, Padua, Italy

**Keywords:** photodynamic therapy, theranostics, graphene, MXene, nanomedicine

## Abstract

Cancer is one of the leading causes of death in the world. Therefore, the development of new advanced and targeted strategies in cancer research for early diagnosis and treatment has become essential to improve diagnosis outcomes and reduce therapy side effects. Graphene and more recently, MXene, are the main representatives of the family of two-dimensional (2D) materials and are widely studied as multimodal nanoplatforms for cancer diagnostics and treatment, in particular leveraging their potentialities as photodynamic therapeutic agents. Indeed, due to their irreplaceable physicochemical properties, they are virtuous allies for photodynamic therapy (PDT) in combination with bioimaging, photothermal therapy, as well as drug and gene delivery. In this review, the rapidly progressing literature related to the use of these promising 2D materials for cancer theranostics is described in detail, highlighting all their possible future advances in PDT.

## Introduction

Photodynamic therapy (PDT) is a form of phototherapy aimed at achieving cell death via the generation of cytotoxic reactive oxygen species (ROS). Although PDT is still an emerging therapeutic modality, it has already been established as a clinically approved method for the treatment of various malignant diseases, including cancer (Agostinis et al., 2011).

Clinically, PDT is usually used in conjunction with other forms of treatments, such as surgery, radiotherapy (RT), and chemotherapy (CT). Due to its local activation and limited tissue penetration, PDT has relatively low invasiveness, and in many cases, good cosmetic results. Therefore, this therapy is particularly suitable for the treatment of exposed skin and sensitive areas, like the head and neck. Moreover, even though it may induce prolonged periods of skin photosensitivity, during which patients need to avoid light, it lacks the serious adverse events (AE) seen in RT and systemic CT. Surgery represents the first-choice treatment and, for the majority of tumor types, the only curative intervention for early diagnosed cancer. However, since most patients are usually diagnosed at late stages, treatments such CT and RT are then preferred. In case of inoperable disease and failure or refusal of other treatments, PDT can potentially be used as a standalone treatment or in combination with other therapies due to the absence of systemic effects and its ability to preserve the organ function. Furthermore, unlike RT, PDT mechanisms of action allow its use also for repeated treatments.

Currently, there are 563 registered clinical trials for PDT, of which almost 60% are directed against cancer (www.clinicaltrials.gov). Among the drug-device combination products, PDT was the first one approved by the Food and Drug Administration (FDA), being now under investigation in preclinical studies to improve its efficacy and safety (Ferreira Dos Santos et al., 2019). The PDT procedure requires three main components: a photosensitizer (PS), a light source (laser), and molecular tissue oxygen. In the context of cancer treatment, the PS is administered locally or systemically, being accumulated in the tumor site. Subsequently, the patient is locally irradiated with light of a proper wavelength, with the aim to activate the PS in the presence of molecular oxygen (Dolmans et al., 2003).

Following administration, PSs can be internalized by both cancer and normal cells. While healthy tissues can eliminate the PS over time, this is not possible for tumor cells due to the lymphatic inadequacy. The resulting PS retention in tumor tissues, together with the localized activation by irradiation, makes PDT a selective treatment for cancer.

The treatment will result in localized oxidative photodamage, consisting in the oxidation of a large range of cellular biomolecules, including nucleic acids, lipids, and proteins. Consequently, the process will lead to selective cytotoxicity, mainly due to a severe alteration in cell signaling cascades and gene expression regulation. The cellular response to photodamage is closed related to several factors. Due to its characteristics, a PS will usually accumulate toward different cellular organelles (e.g., mitochondria and lysosomes), plasma membrane, Golgi apparatus, or endoplasmic reticulum (ER). Generally, three main mechanisms of photodamage-induced cell death have been described: apoptosis, necrosis and autophagy at the tumor site (Bacellar et al., 2015; Ferreira Dos Santos et al., 2019). This process is also accompanied by the induction of an acute local inflammatory reaction that participates in the removal of dead cells, restoration of normal tissue homeostasis and development of systemic immunity (Henderson et al., 2004; Korbelik, 2006). This ability of PDT to activate multiple cell death pathways enables circumvention of apoptosis-resistance in cancer cells, one of the main problems for anticancer approaches.

**Figure 1:**
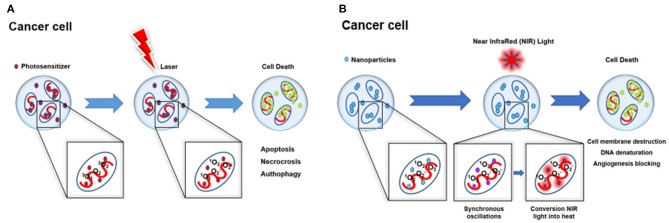
Schematic illustration of phototherapy. **(A)** PDT mechanisms of action and subsequent induced cell death: apoptosis, necrosis, and autophagy. **(B)** PTT mechanisms of action and subsequent cell death induced by cell membrane destruction, DNA denaturation, and angiogenesis blocking.

The superoxide anions released in type I reactions do not pose particular harm to biological systems directly but contribute to the production of hydrogen peroxide, resulting in lipid peroxidation, ultimately leading to the disruption of cellular membranes. Thanks to the short singlet oxygen (^1^O_2_) lifetime of ~40 ns and its short-range action (maximum action radius of about 20 nm), together with the localized PS light-induced activation, PDT is a highly controllable and specific therapy. PS localization can also modulate the subcellular site of action of PDT. Extensive cell damages could also affect apoptotic pathway components, and therefore apoptosis may not be properly executed. Thanks to the autophagy process, cells have the ability to recycle damaged cytoplasmic components and organelles trough the creation of the “autophagosome,” a double membrane structure that after the engulfment of the damaged particles fuses with lysosomes in order to degrade its contents. This autophagic process is not only considered to be a cytoprotective mechanism, being observed also as a cell death mechanism in response to PDT. When the apoptotic mechanism is compromised, cell death mainly occurs through autophagy. This seems to be also correlated with PDT dose, since autophagy can serve as a protective mechanism or initiate the autophagic cell-death, when using low or high doses, respectively (Figures 1A,B). Several preclinical studies have been performed to improve the safety and efficacy of PDT, as well as to extend the number of the different types of diseased tissues that can be treated, thanks to the use of next-generation PSs. The design of second-generation PSs was aimed to develop new agents with higher absorption wavelengths, enabling deeper organs to be targeted thanks to enhanced penetration of light (Lou et al., 2004; Agostinis et al., 2011; Story et al., 2013). Later, the introduction of third-generation PSs allowed improving targeting strategies, such as antibody-directed PS and PS-loaded nanocarriers (Agostinis et al., 2011; Yoon et al., 2013).

Thanks to the progress of nanotechnology, the improvement of PDT using theranostic two-dimensional (2D) nanomaterials (NMs) is attracting growing attention. Therapeutic strategies were combined with imaging modalities for a theranostic aim in order to monitor the biodistribution of therapeutic agents and to identify and/or localize the tumor mass and its growth (Cho et al., 2013; Wang et al., 2013; Ge et al., 2014; Gollavelli and Ling, 2014; Rong et al., 2014; Kim et al., 2015; Wu et al., 2015; Yan et al., 2015b; Guan et al., 2016; Kalluru et al., 2016; Luo et al., 2016; Gulzar et al., 2018).

Currently, multiple combinations of various therapeutic and diagnostic modalities are adopted to achieve a theranostic effect (Orecchioni et al., 2015; Ji et al., 2019), and can be further improved and expanded thanks to the development of NM-based theranostic nanoplatforms. Among 2D NMs suitable for this purpose, graphene and graphene-based materials (GBMs), including few layer graphene (FLG), graphene oxide (GO), reduced graphene oxide (rGO), nano-graphene oxide (NGO), and graphene quantum dots (GQDs), bring the technological innovations needed to the current societal and industrial challenges (Boukhvalov and Katsnelson, 2008; Park and Ruoff, 2009; Gao et al., 2010; Kuila et al., 2012; Mao et al., 2012; James and Tour, 2013; Quintana et al., 2013; Yang et al., 2013a; Roppolo et al., 2014; Sechi et al., 2014; Servant et al., 2014; Kim et al., 2016; Shin et al., 2016; McManus et al., 2017; Park et al., 2017). Graphene, consisting of a single layer of carbon atoms arranged in a honeycomb structure, exhibits a unique combination of physiochemical properties, including high surface area (2,630 m^2^ g^−1^), optimal thermal conductivity (~5,000 Wm K^−1^), remarkable optical transparency (single layer graphene absorbs ~2.3% of visible light), strong mechanical strength (Young's modulus of ~1 TPa), and room temperature quantum hall effect for electrons and holes (Novoselov et al., 2012). Its 2D plane sp^2^ hybridization results in delocalized out of plane π bonds providing an outstanding carrier mobility (ranging from ~ 200,000 to ~500,000 cm^2^ V^−1^ s^−1^, in case of suspended graphene or graphene-based field effect transistors, respectively). Due to its characteristics, graphene offers new fascinating perspectives in nanomedicine for the development of new therapeutic delivery approaches, imaging strategies, as well as biosensor-based diagnostic tools (Yang et al., 2013b; Orecchioni et al., 2014, 2015, 2016b; Avitabile et al., 2018; Fadeel et al., 2018b).

Recently, other promising 2D NMs have attracted attention for their possible applications in various fields, including biomedical sciences (Chen et al., 2015; Luo et al., 2018). One of the most recently discovered 2D materials is MXene, which was first introduced by Gogotsi et al. in 2011 (Naguib et al., 2011). Since then, more than 20 species of MXenes have been successfully synthesized, and the structure/properties of more than 70 have been predicted *in silico*. MXenes are composed of early-transition-metal carbides, carbonitrides, and nitrides with structural formula Mn + 1Xn, where M is an early transition metal, X stands for carbon, nitrogen, or both, and *n* = 1–3. MXenes are synthesized through selectively etching the A-group element from the precursor ternary-layered carbides of MAX phases, where A represents a group of 12–16 periodic table elements. As a consequence of the selectively etching of the A group with -F containing etchants, such as HF, the resulting MXenes will be characterized by abundant surface-terminating functional groups, e.g., hydroxyl (–OH), oxygen (–O), or fluorine (–F), endowing their hydrophilic nature and allowing their flexible surface modification and functionalization. Thanks to the production scalability, the rich surface chemistry, the metallic conductivity, the excellent mechanical/thermal properties, and ease of processability, MXenes have attracted increasing attention for a number of different applications, such as energy storage (Lukatskaya et al., 2017), electromagnetic interference shielding (Shahzad et al., 2016), electrocatalysts (Seh et al., 2016), electrochemical supercapacitors (Ghidiu et al., 2015), and Li-ion batteries (Er et al., 2014; Anasori et al., 2017), just to name a few.

In recent years, MXenes have also been explored for their applications in biomedicine, especially as building-blocks in nano-biotechnology platforms. From the topological perspective, MXenes share all the advantages of other classes of 2D NMs, stemming from their impressive properties, such as extreme thinness, high surface-to-volume ratio, and mechanical toughness. Additionally, the rich chemistry on the surface of MXenes provides abundant reactive sites for enzyme or drug functionalization, while their volumetric capacitance and metallic conductivity are highly desirable for low-noise and high-fidelity biosensors (Driscoll et al., 2018). MXenes exhibit strong absorption in the near-infrared (NIR) region, both in the first (650–950 nm) and second biological window (1,000–1,350 nm), where the low scattering and energy absorption allow maximum penetration of the radiation through the tissue.

The suitability of GBMs for multiple cancer theranostic applications is due to their unique intrinsic physicochemical properties, making them superior nanotools compared to the existing materials and devices used for this purpose, such as optical transparency, high surface area, easy surface functionalization, and low-cost production. In this contest, the use of GBMs and MXenes has been proposed to enhance PDT efficiency. For example, these promising materials are able to correct some of the limits showed by the conventional PSs required for this medical technique. Those are mainly represented by porphyrin-based molecules, such as Chlorin e6 (Ce6), which are characterized by low solubility, photostability, difficulties in delivery efficiency, and inability to be absorbed in regions where the skin is the most transparent (Detty et al., 2004; Huang, 2005). Besides providing a superior biocompatibility, 2D NMs, and in particular GO, can endow them with higher water dispersibility (Gao et al., 2004; Michalet et al., 2005; Resch-Genger et al., 2008), photostability, cytotoxicity, and ROS-generation efficiency (Ge et al., 2014; Pelin et al., 2018). Other materials, such as GQDs, are able to perform better than conventional PDT agents due to their extremely high ^1^O_2_ quantum yield, GQDs (Ge et al., 2014).

Moreover, the particular nanostructure and the large surface area of these 2D NMs facilitate the loading of PSs and other targeting moieties or drugs, allowing a specific release of the treatment and selectivity for cancer cells. Indeed, the presence of the 2D surface characterized by delocalized π electrons and, in particular for GO, the existence of polar functionalities (e.g., epoxide, carbonyl, carboxyl, and hydroxyl groups), allows high drug loading ratios to be reached simply, even of poorly soluble chemotherapeutic drugs, based on electrostatic or hydrophobic interactions and π-π stacking capability, which can even achieve 200 wt% (Augustine et al., 2017). In addition, thanks to the high surface-to-volume ratio, it is possible to reach a superior bio-functionalization, which allows several drugs and molecules to be added, including such fluorescent probes, genes, and targeting moieties to specifically recognize cancer cells, making it possible to achieve their guided and controlled release to the targeted cells.

Furthermore, thanks to the intrinsic NIR absorption properties, GO is a suitable tool for both PDT and photothermal therapy (PTT), obtaining a higher therapeutic efficiency through both *in situ* production of ROS and tumor ablation under NIR irradiation. Together with PDT, PTT represents an alternative anticancer therapy thanks to the selectivity of the hyperthermic process toward cancer cells, sparing healthy tissues. Irradiation of plasmonic NPs accumulated in the tumor with a light of appropriate wavelength leads the NP conduction band electrons to undergo synchronized oscillations, allowing the conversion of NIR light into heat. There are three mechanisms that lead to cell death: cell membrane damage, denaturation of DNA, and angiogenesis blocking (Figure 1B). The investigation of MXenes as PSs for PDT is still in its infancy and, as of June 2019, most of the published works have reported on different MXene species as photothermal conversion agents (PTAs) for PTT (Lin et al., 2016, 2017; Dai et al., 2017; Han et al., 2018; Feng et al., 2019). Indeed, MXenes show higher photothermal effect compared to GO; thus, they appear particularly suitable as PTA for cancer therapy and imaging (Lin et al., 2016, 2018).

**Figure 2:**
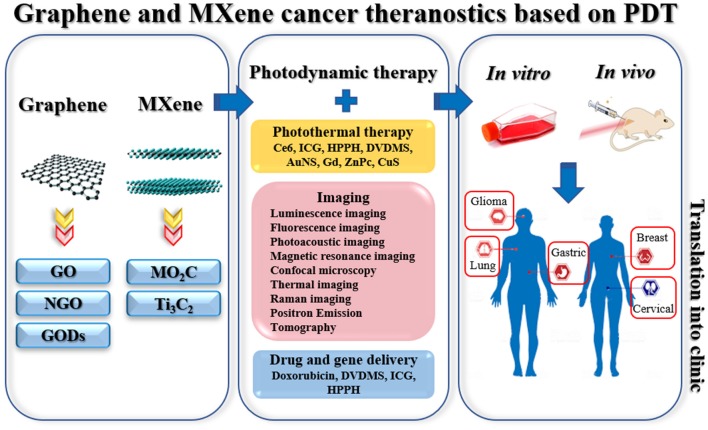
Schematic representation of the current applications in PDT for cancer theranostics based on graphene and MXene. Left panel: representation of graphene and MXene. Middle panel: combined applications with PDT, types of conjugated molecules (for PTT), types of imaging, and examples of conjugated drugs (for drug delivery). Right panel: types of cancer investigated *in vitro* and *in vivo*.

In light of this consideration, in this review, we aim to discuss the current state of the art of PDT in cancer theranostics based on GBMs and MXenes, alone or in combination with other therapies (i.e., PTT and drug delivery). A literature mining protocol was developed to present an overview of the literature in this context, focusing on the different types of models, cancer, functionalization, and combined approaches. A schematic representation of graphene- and MXene-based PDT for cancer theranostic applications is shown in Figure 2. We then have analyzed the future trends in PDT related to graphene and MXene, identifying different knowledge gaps in the field.

## Graphene and MXene Literature Surfing

A systematic review of the literature on graphene and MXene, studied in biomedicine as nanotools for cancer theranostic applications based on PDT, was performed with no time restriction, according to the Preferred Reporting Items for Systematic Reviews and Meta-Analyses (PRISMA) guidelines. The electronic databases (PubMed, Scopus, and ToxLine) were used as data sources, via the following keywords in several different combinations: graphene, GBMs, FLG, GO, rGO, NGO, GQDs, MXene, theranostic, and PDT. To help the reader, Table 1 shows all the acronyms used in the text. As an additional tool, high-impact review articles were also considered. The list of reported studies includes all the retrieved publications from 2008 to January 2019. The adopted inclusion criteria were as follows: (1) studies published in English; (2) full text articles; and (3) the use of PDT in combination with other graphene/MXene applications. A total of 20 eligible studies were identified through the literature review for inclusion in the current review, 1 for MXenes and 19 for GBMs. The latter are summarized in Table 2 based on the different types of cancer, combined applications, species of investigation, model, and material used in the respective study. The trend from 2011 to 2018 displays a remarkable growing interest in graphene and GBMs for cancer PDT.

**Table 1:** List of abbreviations.

**ABBREVIATIONS**	
AE	Adverse events
Ce6	Chlorin e6
CLI	Cerenkov luminescence imaging
CLSM	Confocal laser scanning microscopy
CT	Chemotherapy
DOX	Doxorubicin
DPBF	1,3-diphenyli-sobenzofuran
DVDMS	Sinoporphyrin sodium
EPR	Enhanced permeability and retention
ER	Endoplasmic reticulum
FDA	Federal Drug Administration
GBMs	Graphene based materials
GO	Graphene Oxide
GQDs	Graphene quantum dots
HA	Hyaluronic acid
HB	Hypocrellin B
hMPO	human myeloperoxidase
HPPH	3-(1′-hexyloxyethyl)-3-devinyl pyropheophorbide-a
H_2_O_2_	Hydrogen peroxide
ICG	Indocyanine green
IRT	Infrared thermal imaging
LSPR	Localized Surface plasmon resonance
MIRIBEL	Minimum Information Reporting in Bio–Nano Experimental Literature
miRNA	MicroRNA
MRI	Magnetic resonance imaging
NGO	Nanographene oxide
NIR	Near infrared
NMs	Nanomaterials
^1^O_2_	Singlet oxigen
PAI	Photoacoustic imaging
PDT	Photodynamic therapy
PEG	Polyethylene glycol
PEI	Polyethylenimine
PET	Positron emission tomography
PRISMA	Preferred Reporting Items for Systematic Reviews and Meta-Analyses
PS	Photosensitizer
PTA	Photothermal conversion agents
PTT	Photothermal therapy
rGO	Reduced graphene oxide
RT	Radiotherapy
siRNA	Short interfering RNA
UCL imaging	Upconversion Luminescence Imaging
UCNPs	Upconversion nanoparticles
WHO	World health organization
ZnPc	Phthalocyanine

**Table 2:** Table showing all the studies using GBMs for PDT theranostic applications.

**References**	**Type of cancer**	**Type of applications**	**Model**	**Drug/PS**	**Imaging**	**Material**
Tian et al. (2011)	Cervical cancer	PDT and drug delivery	*In vitro*	Chlorin e6	–	GO-PEG
Huang et al. (2011)	Gastric carcinoma	PDT and drug delivery	*In vitro*	Chlorin e6	–	FA-GO-Ce6
Zhou et al. (2012)	Lung cancer	PDT and drug delivery	*In vitro*	Hypocrellin A and Camptothecin	–	rGO
Wang et al. (2013)	Papilloma, cervical cancer	Imaging, PDT, and PTT	*In vitro and in vivo*	Doxorubicin	CLSM and MRI	UCNPs-NGO/ZnPc
Sahu et al. (2013)	Cervical cancer	PDT and PTT	*In vitro and in vivo*	Methylene blue	–	GO
Cho et al. (2013)	Lung cancer	Imaging, PDT, and PTT	*In vitro*	Chlorin e6	NIR fluorescence imaging	GO–HA–Ce6
Rong et al. (2014)	Breast cancer	Imaging and PDT	*In vitro, in vivo, and ex vivo*	HPPH	PET imaging, (NIR) fluorescence imaging	GO-PEG-HPPH
Zhou et al. (2014)	Lung cancer	PDT and drug delivery	*In vitro*	Hypocrellin A and Camptothecin	–	HA/SN-38/GO
Gollavelli and Ling (2014)	Cervical cancer	Imaging, PDT, and PTT	*In vitro*	–	Fluorescence imaging and MRI	MFG (magnetic and fluorescent graphene)
Ge et al. (2014)	Cervical, breast cancer	Imaging and PDT	*In vitro and in vivo*	–	Fluorescence imaging	NGs-QDs
Yan et al. (2015a)	Lung cancer	Imaging, PDT, and PTT	*In vitro and in vivo*	DVDMS	Fluorescence imaging and PAI	GO-PEG-DVDMS
Wu et al. (2015)	Breast cancer	Imaging, PDT, PTT, and drug delivery	*In vitro*	Indocyanine green	NIR fluorescence imaging	pGO-CuS/ICG
Yan et al. (2015b)	Brain cancer	Imaging, PDT and drug delivery	*In vitro, in vivo, and ex vivo*	DVDMS	Fluorescence imaging	GO-PEG-DVDMS
Kim et al. (2015)	Cervical cancer	Imaging, PDT, and PTT	*In vitro*	Au	Raman Bioimaging	PEG-Au@GON NPs
Luo et al. (2016)	Lung cancer	Imaging, PDT, and PTT	*In vitro and in vivo*	–	Fluorescence confocal microscope NIR fluorescence and thermal imaging	NGO-808
Kalluru et al. (2016)	Melanoma	Imaging, PDT and PTT	*In vivo*	–	Fluorescence imaging	GO-PEG-folate
Wo et al. (2016)	Esophageal squamous carcinoma	PDT, PTT, drug delivery, and magneto-mechanical therapy	*In vitro*	Doxorubicin	–	HMNS/SiO2/GQDs-DOX
Wu et al. (2017)	Breast cancer	Imaging, PDT, and PTT	*In vitro and in vivo*	Chlorin e6	CLSM, thermal/PT imaging	GO/AuNS-PEG and GO/AuNS-PEG/Ce6
Gulzar et al. (2018)	Liver and cervical cancer	Imaging, PDT, and PTT	*In vitro, in vivo, and ex vivo*	Chlorin e6	UCL imaging	NGO-UCNP-Ce6 (NUC)

*The 19 selected studies were characterized on the basis of different type of cancer, application, model, drug/PS, imaging method and material*.

A search on clinical trials was performed using the same criteria; however, although there are currently 325 clinical trials based on PDT for cancer therapy (www.clinicaltrials.gov), none of them involves GBMs or MXenes. This result highlights that the research on 2D nanomaterials for PDT, despite promising results obtained *in vitro* and *in vivo*, is still at a very early stage for a clinical translation.

**Figure 3:**
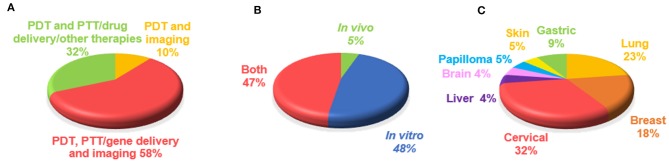
Overview of graphene-based PDT theranostics. Percentages of manuscripts (19 papers) on the basis of **(A)** type of applications combined with PDT, **(B)** model used for the study (*in vivo* or *in vitro*), **(C)** type of cancer studied.

The 19 manuscripts on graphene-based PDT in cancer theranostics were analyzed with respect to type of applications combined with PDT, model used for the study (*in vivo* or *in vitro*), and type of cancer studied (Figure 3). Focusing on the association of other cancer theranostic applications, it emerged that PDT was often applied together with one or multiple therapies and imaging modalities: the majority of the studies (58%) concerned the simultaneous application of PDT, imaging, and PTT, drug delivery or other therapies, followed by the combination of PDT with PTT, drug delivery or other therapies (32%), while only 10% of the works used PTD associated with imaging alone (Figure 3A). In particular, PDT was used in combination with different imaging techniques, such as luminescence imaging (CLI), fluorescence imaging, photoacoustic imaging (PAI), magnetic resonance imaging (MRI), confocal microscopy (CLSM), thermal imaging (IRT), Raman imaging, and positron emission tomography (PET). From the analysis of the model used in the works, it emerged that most studies were carried out both *in vitro* and *in vivo* (48%), a large number (47%) using only *in vitro* models consisting of different kinds of cancer cells, while only 5% tested these materials exclusively *in vivo* (Figure 3B).

Finally, we focused on the different types of tumors studied (Figure 3C), identifying cervical cancer as the most investigated (32% of publications). Indeed, according to the World Health Organization (WHO)^1^, cervical cancer is the fourth most common type of tumor in women and the eighth most frequently occurring overall; rising with 570,000 new cases in 2018 and representing 6.6% of all female cancers (from world health organization www.who.int). The second largest portion comprises works focusing on lung cancer (23%), followed by publications concerning breast cancer (18%) and gastric cancer (9%). Papers investigating other kinds of tumors, such as skin, brain, liver cancer, and papilloma, make up the remainder (5%).

## Progress in Photodynamic Therapy in Graphene- and MXene-mediated Theranostics

GBMs have attracted attention for PDT exploiting their optical loading properties (Avitabile et al., 2018; Viseu et al., 2018). Studies in the field of theranostics started by using GBMs as delivery vehicles for both PS and imaging agent (Sahu et al., 2013; Gollavelli and Ling, 2014), paving the way to the following research for a more detailed exploration of nanotechnology-based PDT in cancer theranostics. Graphene has been shown to adsorb light in the near infrared (NIR) region, allowing its potential application for cancer phototherapy to be evaluated both *in vivo* and *in vitro* (Cho et al., 2013; Sahu et al., 2013; Wang et al., 2013; Gollavelli and Ling, 2014; Rong et al., 2014; Kim et al., 2015; Wu et al., 2015, 2017; Yan et al., 2015a; Kalluru et al., 2016; Luo et al., 2016; Wo et al., 2016; Gulzar et al., 2018).

Moreover, it has been demonstrated, both in cell and animal models, that GBMs exhibit several advantages for drug delivery, giving the possibility of high drug loading efficiency, controlled drug release and tumor-targeted drug delivery (Bitounis et al., 2013; Yang et al., 2016; Zhang et al., 2016, 2017). Indeed, different biomolecules, such as DNA, microRNA (miRNA), short interfering RNA (siRNA), and chemotherapeutic drugs can be loaded onto the surface of these materials for gene transfection and drug delivery (Huang et al., 2011; Tian et al., 2011; Zhou et al., 2014; Yan et al., 2015b; Wo et al., 2016; Wu et al., 2017). GBMs and MXenes are also suitable for imaging purposes. In particular, GO-based nanoplatforms show great potential exploitable for imaging purposes, thanks not only to the efficient quenching properties of GO toward several fluorescent moieties, including dyes, quantum dots, and conjugated polymers, but also to its ability to improve their stability, distribution, biocompatibility, and photodynamic efficiency (Yan et al., 2015b). Other materials, such as NGO sheets, have a photoluminescent emission in the visible and infrared regions (Sun et al., 2008). This intrinsic photoluminescence (PL) can be exploited for little background-NIR live cell imaging (Sun et al., 2008). GQDs exhibit multiple properties, ranging from their broad absorption in the visible and NIR light range, their good aqueous dispersibility, deep-red emission, high pH and photo-stability up to their positive biocompatibility. In addition, GQDs display a relevant ^1^O_2_ generation yield, beyond 1.3 (almost double compared to the other PDT agents studied in literature). Among the various unique properties, GQDs also present an up-conversion PL (Zhu et al., 2012a,b; Feng et al., 2017), ranging from blue to yellow colors (Li et al., 2013). Due to all their properties, GQDs are able to behave as a multifunctional nanoplatform for the theranostic combination of imaging and highly efficient *in vivo* PDT (Ge et al., 2014).

**Figure 4:**
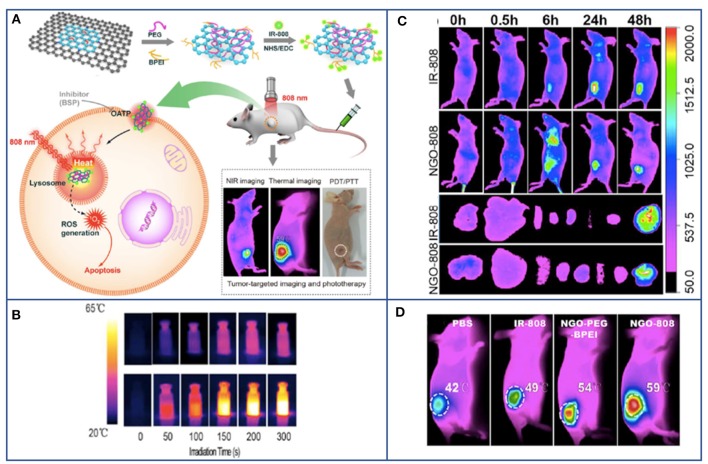
Example of PDT based on graphene for combined and multimodal applications in cancer theranostic. **(A)** Schematic illustration of NGO-808 preparation and combined A549 tumor xenografts-targeted NIR imaging and synergistic phototherapy (PDT and PTT). **(B)** Thermal images showing the higher heat generation of NGO-808 (bottom row) compared to that in blank phosphate-buffered saline (upper row) during 5 min 808 nm laser irradiation. **(C)** *In vivo* NIR imaging of NGO-808 on A549 tumor xenografts. **(D)** *In vivo* combined PDT and PTT on A549 tumor xenografts treated with NGO-808. Adapted with permission from Luo et al. (2016), copyright 2016 American Chemical Society.

For these reasons, scientists attempted to exploit these therapeutic and imaging potentialities of GBMs in cancer theranostics to achieve targeted cancer cell killing as well as less impairment of healthy cells. An example of PDT based on graphene for combined applications in cancer theranostic is reported in Figure 4.

### Combined Therapy: PDT and Drug Delivery/PTT

The development of outstanding nanoplatforms leveraging PDT and synergistic therapies, based on drug delivery and PTT, is currently being extensively investigated for cancer treatment. In one of the earliest works showing the promise of GBMs in PDT, in 2011, Tian et al. loaded polyethylene glycol (PEG)-functionalized GO with the PS Ce6 *via* supramolecular π-π stacking (Tian et al., 2011). The material was taken up by cervical cancer cells and resulted in the formation of ROS under light excitation. Anti-cancer activity of the GO-PEG-Ce6-mediated PDT protocol was more pronounced compared to free Ce6. Also, Huang et al. proposed to use GO as a delivery platform for Ce6 (Huang et al., 2011). Like the above studies, Ce6 was loaded onto folic acid targeted GO through π-π stacking and hydrophobic interactions. The system was shown to kill MGC803 gastric cancer cells upon irradiation. Later, Zhou et al. efficiently loaded GO with the PS hypocrellin B (HB) through π-π stacking interaction. They showed that the material was able to generate ^1^O_2_ upon irradiation (Zhou et al., 2012). The same group later reported that the efficiency of PS-loaded GO anticancer activity could be even improved through its combination with chemotherapy (Zhou et al., 2014). In particular, in this study, hypocrellin A and 7-ethyl-10-hydroxycamptothecin were co-loaded on GO and the resulting system induced higher cell death in a lung cancer cell line model when exposed to light, demonstrating that chemotherapy and PDT can work synergistically (Zhou et al., 2012).

Later, in another study, functionalized nano-graphene oxide (NGO) was complexed with a PS methylene blue in order to achieve combined PTT/PDT of cancer (Sahu et al., 2013). Due to the pluronic functionalization, material showed great stability in biological fluids. Authors reported that the nanocomplex was efficiently taken up by cancer cells and able to release methylene blue in a pH-dependent manner. Only when exposed to light did the system showed anti-cancer activity *in vitro*. Following its systemic administration in tumor bearing mice, nanocomplex was shown to accumulate in tumor. When mice were irradiated with NIR light, it caused total ablation of tumor tissue through the combined action of photodynamic and photothermal effects.

These studies suggested the possibility of exploiting the properties of GBMs and MXene to perform an improved PDT in multimodal nanosystems for cancer treatment and paved the way up for future theranostic works.

### Theranostics: Imaging and PDT

GBMs can be used not only in PDT protocols and other combined therapies, but also for imaging purpose, allowing the development of new theranostic nanoplatforms for cancer diagnosis and treatment. For example, in 2014, Ge et al. exploited the intrinsic properties of graphene quantum dots (GQDs), such as the broad absorption from the visible to the NIR, high pH- and photo-stability, and biocompatibility for imaging purposes (Ge et al., 2014). In this study, GQDs exhibited a massive ^1^O_2_ generation yield, making them efficient multifunctional nanoplatforms for *in vivo* simultaneous imaging and extremely efficient PDT of different types of cancer, including skin melanoma and tumors located near the skin. During the same year, Rong et al. identified PEG-functionalized GO as a suitable nanoplatform to increase PDT efficacy and improve long-term survival after treatment (Rong et al., 2014). This was obtained mainly thanks to the ability of the GO-based nanotool to serve as a carrier for the PS agent HPPH, increasing its accumulation to the cancer site. In their *in vivo* studies, the distribution and delivery were traced through fluorescent imaging and positron emission tomography (PET) by the ^64^Cu radiolabeling of HPPH. Compared to free HPPH, GO-PEG-HPPH enhanced the photodynamic cancer cell killing ability thanks to HPPH's improved tumor delivery.

### Theranostics: Imaging, PDT, and Drug Delivery

In a study directed against lung cancer, a novel photo-theranostic platform based on sinoporphyrin sodium (DVDMS) loaded on PEGylated GO was investigated (Yan et al., 2015a). The GO-PEG carrier improved the loaded PS DVDMS fluorescence through intramolecular charge transfer and facilitated tumor accumulation efficiency of DVDMS by enhanced permeability and retention effect. The NIR absorption of GO was enhanced by DVDMS, leading to improved photoacoustic imaging and PTT. The *in vivo* intravenous injection of low doses of GO-PEG (1 mg/kg) and of DVDMS (2 mg/kg) resulted in 100% tumor eradication.

### Theranostics: Imaging, PDT, and PTT

New advances have been made in cancer treatment to establish a targeted protocol that covers the simultaneous application of imaging methods for diagnosis and PTT or PDT for its care. The theranostic progress made by the early studies led to the development of new combined protocols involving graphene nanoplatforms for a simultaneous imaging, PTT, and PDT approach. Wang et al. developed a promising integrated probe for UCL image-guided conjunctional PDT/PTT of cancer (Wang et al., 2013). This multifunctional nanoplatform (UCNPs-NGO/ZnPc) was composed of covalently grafted core–shell structured upconversion nanoparticles (UCNPs) with nanographene oxide (NGO) via bifunctional PEG loaded with phthalocyanine (ZnPc). Authors suggested that this nanoplatform could be used as UCL high contrast imaging probing of cells and whole-body for diagnosis, as well as for PDT causing the formation of cytotoxic ^1^O_2_ under light excitation and for PTT, by converting the 808 nm laser energy into thermal energy (Wang et al., 2013). Another promising platform for combined PTT/PDT directed for lung cancer was realized by combining biocompatible HA-conjugated Ce6 with GO (Cho et al., 2013). This dual PTT/PDT enzyme-activatable GO–PS nanoplatform (GO–HA–Ce6) acts as a biologically tunable agent, exploitable for NIR fluorescence imaging and photo-induced cancer therapy (Cho et al., 2013). Another incredible example of graphene-based combined multimodal nanosystem for simultaneous imaging, NIR-induced PTT and PDT was presented by Wu et al. (2017). In this research, all the promising applications of graphene/Au-based nanohybrids have been summed up into one single nanoplatform. They formulated a graphene-Au nanostar hybrid NM (GO/AuNS-PEG) activated by a single wavelength laser-mediated phototheranostic design, based on the loading of Ce6 (GO/AuNS-PEG/Ce6) (Sahu et al., 2013). Gollavelli et al. developed a superparamagnetic graphene-based nanoplatform, so-called MFG-SiNc4, carrying the hydrophobic silicon napthalocyanine bis (trihexylsilyloxide) (SiNc4) PS (Gollavelli and Ling, 2014). The graphene used in the study showed a wide range for NIR absorption (600–1,200 nm). Therefore, the presence of SiNc4, working at any wavelength within this range, facilitated the possibility of single light induced phototherapy. *In vitro* and *in vivo* results have shown that the simultaneous dual modal imaging and PTT/PDT abilities of magnetic fluorescent graphene MFGeSiNc4 achieved a significant cell killing efficacy which was a synergistic effect of PDT and PTT. In 2016, Kalluru et al. reported for the first time that NGO showed single-photon excitation wavelength-dependent photoluminescence in the visible and short NIR region, which could be exploitable for *in vivo* multi-color fluorescence imaging (Kalluru et al., 2016). NGO induced the formation of ^1^O_2_ both *in vitro* and *in vivo* for combined PDT and PTT in melanoma. When mice with B16F0 melanoma tumors were irradiated with NIR light at ultra-low doses, their average half-life span was improved. In another study, with the introduction of PS (IR-808) on nanoGO, authors were able to combine NIR imaging synergistically with enhanced PDT and PTT (Luo et al., 2016). Tumors were treated with PEG- and PEI-functionalized NGO-808 and irradiated; apoptosis and necrosis occurred, obtaining as result the complete ablation of the tumor. Moreover, no recurrence was observed after 60 days post-irradiation.

In various studies, PEG was combined with the graphene-based nanoplatforms to improve PDT efficacy or the material biodistribution. For example, in 2015, Kim et al. studied the effects of PEGylated graphene-gold nanoparticles (ZnPc-PEG-Au@GON NPs) that, beside possessing a photothermal effect, positively displayed multiple roles as Raman imaging agents, delivery vehicle of ZnPc, and PS for enhanced combined imaging, PTT, and PDT diagnosis and therapy (Kim et al., 2015). A simultaneous and synergistic combination of PDT and PTT was achieved as well as thermal and fluorescence imaging. GO/AuNS-PEG composite demonstrated to produce a high photothermal conversion efficiency due to the graphene and gold nanostars enhanced optical absorbance in the NIR range. The PS-assembled graphene/gold nanostar hybrid completely eliminated the EMT6 xenografted tumors thanks to the synergistic *in vivo* cancer cell killing of parallel PDT and PTT under a single NIR laser irradiation (660 nm). Indeed, this study further inspires new graphene-Au nanostar hybrid applications as biocompatible nanoplatforms for imaging-guided (fluorescence/thermal/photoacoustic imaging) multimodal breast cancer therapy (PDT/PTT/chemotherapy/sonodynamic therapy) (Wu et al., 2017).

More recently, Zhang et al. (2019) proposed Mo_2_C MXene *ad hoc* synthesized in the nanosphere (NSph) topology as a theranostic nanoagent for combined cancer dual therapy (PTT and PDT) and imaging (photoacoustic and computerized tomography). Also in this work, the ROS generation capability was characterized by the modulation of DPBF absorbance at 420 nm under NIR irradiation at 1,064 nm and confirmed by the inhibition of DPBF degradation upon addition of the ROS quencher NaN_3_. In addition, synergistic PTT and PDT on human liver carcinoma cells (HepG2) *in vitro* revealed more than 80% of apoptotic cells in a dose-dependent manner, confirming the critical contribution of ROS generation to the efficacy of the Mo_2_C-mediated PTT/PDT synergistic therapy. *In vivo* antitumor efficacy tested 14 days post-treatment in HepG2 tumor bearing mice showed complete tumor ablation and lack of regrowth after 10-min NIR exposure in the presence of Mo_2_C pre-injected into the tumor, whereas control animals, either non-irradiated or irradiated without Mo_2_C, showed a 4-fold increase in tumor volume. Hematologic, body weight, and post-mortem histological analysis of explanted organ tissue supported the safety of Mo_2_C NSph as an injectable PTA for cancer theranostics.

### Theranostics: Imaging, PDT, Drug Delivery, and PTT

A low number of studies were carried to evaluate the use of GBMs and MXenes for simultaneous imaging, PDT, drug delivery, and PTT. In 2015, a NIR photo-triggered drug delivery system pGO-CuS/IndoCyanine Green (ICG) exhibited high efficacy of photothermal conversion, being a perfect candidate for highly efficient controlled theranostic applications (i.e., bimodal PDT and PTT therapy plus NIR imaging for a broad range of deep-seated cancer tissues) (Wu et al., 2015). This promising nanoplatform displayed optimal stability, high loading efficiency of ICG, good photon energy conversion to heat and significant ^1^O_2_ generation yield under NIR laser treatment. It is able, via passive transmembrane pathway, to readily reach the cellular inner cytoplasm as a potent synergic platform for PDT and PTT, killing specifically cancer cells by the appropriate tuning the two NIR light irradiations (Wu et al., 2015). Later, Wo et al. developed a multimodal system which was able to enclose four different synergetic anti-cancer activities: photodynamic toxicity, photothermal damage, chemotherapy, and magnetic field-mediated mechanical stimulation (Wo et al., 2016). The authors formed liposome-stabilized doxorubicin (DOX)-loaded magnetic nanospheres, aimed at enhancing anti-cancer activity through magnetic field-mediated mechanical force and NIR laser irradiation.

**Figure 5:**
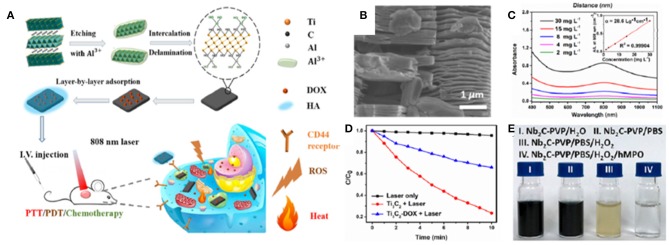
MXenes in cancer PDT. **(A)** Schematics of a multimodal PTT/PDT/chemotherapy synergistic platform based on Ti_3_C_2_ NSs functionalized with DOX. Included is the synthesis if the Ti_3_C_2_ NSs from the precursor Ti_3_AlC_2_ phase, followed by the exfoliation, intercalation and functionalization steps. **(B)** Scanning electron microscopy image of exfoliated Ti_3_C_2_ NSs. **(C)** Absorbance spectra and extinction coefficient at varying concentrations of Ti_3_C_2_ MXene. **(D)** ROS generation under NIR irradiation at 808 nm in the presence of Ti_3_C_2_ and Ti_3_C_2_-DOX NS detected by DBPF absorbance assay. Reproduced with permission from Liu et al. (2017). **(E)** Biodegradation of Nb_2_C NS. Reproduced with permission from Lin et al. (2017).

Liu et al. (2017) first demonstrated the feasibility of Ti_3_C_2_ MXene nanosheets (NSs) as PSs for PDT in a PTT/PDT/chemo-synergistic platform (Figures 5A–C). ROS generation in the presence of Ti_3_C_2_ NSs in aqueous suspensions was investigated using 1,3-diphenyli-sobenzofuran (DPBF) as the singlet ^1^O_2_ detector. Upon NIR irradiation of Ti_3_C_2_ NSs at 808 nm for 10 min, DPBF showed a ~80% decrease in absorbance at 420 nm, consequently revealing the generation of ^1^O_2_ (Figure 5D). Similar ROS generation capability, although less pronounced, was observed when Ti_3_C_2_ functionalized with DOX was exposed to the same irradiation protocol, thus enabling the development of combined PDT/chemotherapeutics. The exact mechanism of ^1^O_2_ generation in Ti_3_C_2_ is still unclear and warrants further investigations. The authors attribute it to the energy transfer of photoexcited electrons from Ti_3_C_2_ to triplet oxygen (ground state oxygen, ^3^O_2_), a mechanism similar to the photodynamic behavior of GQDs (Ge et al., 2014) and black phosphorous (Wang et al., 2015). The localized surface plasmonic resonance (LSPR) effect might also play a role similar to what has been reported for metals, like gold nanoparticles. In these systems, the efficiency of energy transfer is enhanced when the particles are in the aggregated state. Thus, the high surface area of Ti_3_C_2_ might be particularly favorable for LSPR. Compared to the individual therapeutic modalities, synergistic PTT/PDT/chemotherapy with Ti_3_C_2_-DOX led to significant improvements in therapeutic efficacy and recurrence outcomes against human colon carcinoma (HCT-116) *in vivo* in tumor-bearing mice. The abundant surface termination in the Ti_3_C_2_ NSs also enables specific functionalization to selectively target species in the tumor microenvironment. For example, coating Ti_3_C_2_-DOX with hyaluronic acid increased colloidal stability and actively targeted the surface protein CD44^+^ overexpressed in breast cancer cells.

#### Theranostic Outcomes of the *in vivo* Studies

By analyzing the *in vivo* outcomes, it is possible to conclude that all the studies presented very promising results for the use of these materials in cancer theranostics, achieving a significant (Kalluru et al., 2016; Gulzar et al., 2018) or even total cancer ablation without tumor recurrence (Sahu et al., 2013; Wang et al., 2013; Ge et al., 2014; Rong et al., 2014; Yan et al., 2015a,b; Luo et al., 2016; Wu et al., 2017), as shown in Table 3. Among all the materials, GO-based nanoplatforms were the most investigated (5 *in vivo* studies out of 9), followed by NGO and GQDs (3 and 1 studies out of 9, respectively). Since GO and NGO can act both as a photothermal agent and a delivery carrier for PSs, most the works exploited its theranostic potential for combined PDT and PTT. This strategy led to total cancer ablation between 14 and 21 days.

**Table 3:** Comparison of nanomaterials and laser powers used for PDT-based cancer theranostic applications and relative outcomes *in vivo*.

**References**	**Tumor**	**Laser wavelength**	**Laser power intensity**	**Irradiation time**	**Starting material**	**Combined therapy**	**Outcome** **(number of days after the treatment)**
Sahu et al. (2013)	Cervical cancer	650 nm	0.1 W/cm^2^	10 min (one time)	NGO	PTT	Total ablation (15 days)
Ge et al. (2014)	Cervical, breast cancer	400–800 nm	0.1 W/cm^2^	10 min (two times)	GQDs	–	Total ablation (17 days)
Rong et al. (2014)	Breast cancer	671 nm	90.0 W/cm^2^, 0.1 W/cm^2^	20 min (one time)	GO	–	Total ablation (60 days)
Yan et al. (2015a)	Lung cancer	630 nm	16.0 W/cm^2^	5 min (one time)	GO	PTT	Total ablation (14 days)
Yan et al. (2015b)	Brain cancer	630 nm	156.0 W/cm^2^	– (one time)	GO	Drug delivery	Total ablation (10 days)
Luo et al. (2016)	Lung cancer	808 nm	1.0 W/cm^2^	5 min (one time)	NGO	PTT	Total ablation (16 days)
Wu et al. (2017)	Breast cancer	660 nm	0.8, 1.2 and 2.0 W/cm^2^	10 min (one time)	GO	PTT	Total ablation (21 days)
Gulzar et al. (2018)	Liver and cervical cancer	808 nm	0.7 W/cm^2^	10 min (one time)	NGO	PTT	Partial ablation (14 days)
Kalluru et al. (2016)	Melanoma	808 nm	0.2 W/cm^2^	8–10 min (every day)	GO	PTT	Partial ablation (14 days)

Thanks to the to the quantum confinement effect related to their small dimensions, NGO and GQDs possess non-blinking photoluminescence and photostability. Therefore, these materials have been explored mainly for PDT in association with UCL imaging (Luo et al., 2016; Gulzar et al., 2018) or fluorescence imaging (Ge et al., 2014; Luo et al., 2016). In particular, two studies investigated NGO for imaging-guided PDT in combination with PTT (Luo et al., 2016; Gulzar et al., 2018), achieving significant (Gulzar et al., 2018) or even total (Luo et al., 2016) cancer ablation between 14 and 16 days.

Finally, only one study evaluated the suitability of GQDs for PDT-based cancer theranostics based on PDT (Ge et al., 2014). The main advantage of GQDs is represented by their ability to serve as imaging tools and perform better than conventional PDT agents in terms of ^1^O_2_ quantum yield. However, since this result is achieved in the visible light region, the suitability of these tools appears to be limited to superficial tumors, such as skin cancer.

## Future Perspectives

Following its discovery, graphene has attracted attention of the society in general with several expectations from the public in the context of nanomedicine (Sechi et al., 2014), and the turn of MXene is already on the stage.

Thanks to their chemical, physical and biological properties, graphene and MXene have shown to be powerful tools for PDT in cancer theranostics. Both 2D nanosystems allow the simultaneous application of non-invasive bioimaging and therapeutic strategies that can be associated with PDT, including photothermal therapy, magnetic therapy, and remotely controlled chemotherapy by drug and gene delivery.

The suitability of these promising materials as photothermal agents for tumor therapy and imaging is due to their ability to adsorb light in the NIR region. Moreover, the easy functionalization capability, thanks to their high surface-to-volume ratio, allows the loading of photosensitizer agents on these nanoplatforms, enhancing the targeting and efficiency, resulting in a more localized action, characterized by reduced side effects and improved therapy.

In addition, the combination with chemotherapeutic drugs loaded on these 2D NMs can work synergistically with PDT, leading to an improved anticancer activity. Furthermore, the functionalization with other agents, such as Au, endows them with a high photothermal conversion efficiency that can further enhance their optical absorbance in the NIR range. Beside the therapeutic efficiency of these nanoplatforms, their stability in aqueous matrix can also be improved thanks to their ability to be loaded with hydrophilic molecules, such as PEG. This aspect is of great interest in view of their intravenous administration, since most PSs are hydrophobic and could easily aggregate in biological fluids, with a consequent decrease in their quantum yield and increase in immune responses (Sibani et al., 2008; Kashef et al., 2017).

However, despite the encouraging promising results, there is still an extensive work to be accomplished to further clarify and prove the potentialities of GBM- and MXene-based PDT for cancer theranostics. First of all, for the translation of these 2D materials into clinical application, the assessment of their long-term toxicity is required to fully characterize their safety profile.

The potential widespread use of graphene and MXene-based materials for commercial purposes will favor their interactions with biological and environmental components. Therefore, several studies have been carried out to define the cyto- and bio-compatibility of these nanomaterials *in vitro* and *in vivo* (Fadeel et al., 2018a; Lin et al., 2018). These studies, particularly for graphene, state that the toxicity depends on the complex interaction of several physiochemical properties, such as shape, size, functional groups, oxidative state, dispersion state, synthesis methods, exposure times, as well as route and dose of administration. Moreover, graphene can contain several chemical contaminants and impurities coming from synthesis (Liao et al., 2018) and post-synthesis processing steps that can lead to graphene structure disruption and smaller carbonaceous debris production. Therefore, these confounding aspects may elicit variable toxicity responses (Li and Boraschi, 2016).

In this view, various studies have been performed to better understand and predict GBM toxicity and their potential impact on the immune system which governs every aspect of our health, including the way we react to therapies in cancer (Orecchioni et al., 2014, 2016a,b, 2017; Russier et al., 2017; Fadeel et al., 2018b). The obtained results have highlighted the importance of material characterization as a key element for hazard assessment as well as bio and immune compatibility. Therefore, biomedical scientists should not consider graphene as a single material but as a complex class of materials, taking into account the role of physicochemical properties (e.g., lateral dimension, carbon oxygen ratio and number of layers) while assessing the biological effects (Fadeel et al., 2018a). Concerning MXene, preliminary evaluations of Ti_3_C_2_ MXene biocompatibility have not evidenced apoptosis or signs of cytotoxicity *in vitro* in cancer cells (Dai et al., 2017; Lin et al., 2017; Liu et al., 2017; Han et al., 2018) and neurons (Driscoll et al., 2018). Ti_3_C_2_ NSs injected *in vivo* in the blood stream appear to be either excreted in the urine via physiologic renal clearance pathways or retained in the tumor site via the enhanced permeability and retention (EPR) effect, without accumulating in the major organs. Similar findings have been reported for the cyto-biocompatibility and systemic safety of Ta_4_C_3_ (Dai et al., 2017; Liu et al., 2018) and Nb_2_C MXenes (Lin et al., 2017). Despite all these data on MXene biocompatibility, the assessment of its potential toxicity is still at a very early stage. For both types of materials, new studies on their biomedical application should take into consideration general requirements with respect to Minimum Information Reporting in Bio–Nano Experimental Literature (MIRIBEL) and other key considerations on the issue of transparency and reproducibility in nanomedicine, such as that the choice of material physical-chemical characteristics should be tailored for their intended use (Faria et al., 2018; Leong et al., 2019). That consideration is even more valid in the context of cancer theranostics were the starting properties of the material can make the difference in the successful application of new 2D material-based cancer treatment. Another key point is related to the materials' fine characterization and reproducibility, which should be considered in every work based on engineered nanomaterials as a key aspect to avoid hype around the potential translation into clinic (DeLoid et al., 2017).

Beyond the toxicity context and physical-chemical material choice, other knowledge gaps need to be filled to shed light on the actual potentialities of these 2D NMs for cancer theranostics and PDT in particular. Indeed, the understanding of these aspects cannot overlook the elucidation of fundamental mechanisms underlying the ROS generation elicited by these materials, which is at the basis of PDT. Moreover, more efforts should be directed into a deeper understanding of the nanoparticle-tumor interaction, the possibility of a scaled-up synthesis, and the development of regulatory theranostic protocols in order to determine a personalized therapy framework.

Finally, controlling the lifetime of the nanoagents in the body and mitigating the risks related to retention of NMs and their byproducts could significantly advance NM-based theranostic platforms in the translational pipeline (Orecchioni et al., 2015). The complete clearance from the body, along with the biodegradation of 2D nanostructures needs to be assessed for these materials in order to be translated into clinical settings (Andón et al., 2013; Bhattacharya et al., 2013; Farrera et al., 2014; Kurapati et al., 2018; Mukherjee et al., 2018; Martín et al., 2019). Although GBMs can be considered structurally persistent, it has been proved that oxidative enzymes (i.e., peroxidases) are capable of catalyzing the GO degradation *in vitro* and *in vivo* (Bai et al., 2014; Kurapati et al., 2018; Mukherjee et al., 2018). Nb_2_C MXene NSs can be engineered to degrade through an active biodegradation scheme that leverages human myeloperoxidase (hMPO), a free-radical species generating enzyme expressed by neutrophils to carry out their antimicrobial activity (Lin et al., 2017). In the presence of hydrogen peroxide (H_2_O_2_), hMPO generates hypochlorous acid and reactive radical intermediates, which degrade polymers and carbon-based materials. The incubation of Nb_2_C NSs in hMPO and H_2_O_2_ enriched medium for 24 h has been reported to cause the complete degradation and disappearance of NSs, thus demonstrating *in vitro* the feasibility of this enzyme-triggered degradation route for MXenes (Figures 5D,E).

Overall, this review has shown that significant advances in the theranostic use of graphene-based materials and MXenes have been made. However, three main aspects should be carefully taken more into account: (i) the potential not-targeted toxicity, (ii) a choice of physical-chemical material characteristics prior their assessment for cancer therapy, (iii) a fine characterization, and (iv) the assessment of their potential biodegradability. Despite that knowledge gaps in the field still need to be filled, virtuous perspectives for GBMs and MXene were evidenced from over 30 works here analyzed, standing out as the most promising 2D NMs intended to change the patterns of conventional cancer theranostics, guaranteeing new protocols for personalized therapies.

## Author Contributions

LD proposed the topic of the review and designed and coordinated the work. LD, AY, and FV wrote the manuscript with the help from AG, LF, and AK. AG, LF, and AK investigated the literature and prepared the figures with the help of LD, AY, and FV. DB and BZ critically revised the manuscript. All authors discussed and revised the manuscript.

### Conflict of Interest

The authors declare that the research was conducted in the absence of any commercial or financial relationships that could be construed as a potential conflict of interest.

## Footnotes

^1^WHO. *Cervical Cancer WHO*. Available online at: http://www.who.int/cancer/prevention/diagnosis-screening/cervical-cancer/en/ (accessed July 4, 2019).
